# “Umbrella-shaped pulley traction” assisted endoscopic submucosal dissection of colonic lesions – a modified external traction technique

**DOI:** 10.1055/a-2751-9622

**Published:** 2026-01-08

**Authors:** Rui Xie, Ru Feng, Xiaozhong Yang, Honggang Wang, Weijie Dai

**Affiliations:** 1 91596 Department of Gastroenterology, The Affiliated Huai’an No. 1 People’s Hospital of Nanjing Medical University, Huai’an, China; 2 91596 Department of Endoscopy Center, The Affiliated Huai’an No. 1 People’s Hospital of Nanjing Medical University, Huai’an, China

Colorectal endoscopic submucosal dissection (ESD) is technically demanding because of the difficulty in adequately visualizing the submucosal layer. Therefore, building good traction to provide a clear view for ESD is critically important. Gravity, injection fluid, or a clip-with-line approach has been used to optimize traction during ESD to improve performance 1 . However, the traction direction and ability are limited in most traction methods, resulting in insufficient effects in some cases 2 . Therefore, we adopted a new multi-point and multi-directional external traction method (umbrella-shaped pulley traction) to treat a case of extensive laterally spreading tumors (LSTs) in the rectum ( Video 1 ).

“Umbrella-shaped pulley traction” assisted endoscopic submucosal dissection of an extensive LST in the rectum. LST, laterally spreading tumor.Video 1

**Fig. 1:**
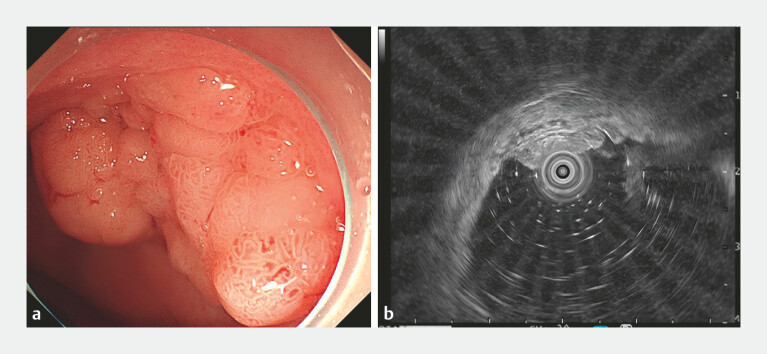
**a** Colonoscopy indicated an LST lesion in the rectum, as a nodular mixed type. **b** The ultrasound endoscope indicates that it originates from the mucosal layer, and the submucosa is intact. LST, laterally spreading tumor.

A 69-year-old woman was found to have a LST (granular type and nodular mixed type) in the rectum. Endoscopic ultrasound indicated that the lesion originated from the mucosal layer and was approximately 2 cm × 3 cm ( Fig. 1 ). Therefore, she received ESD for this lesion.

**Fig. 2:**
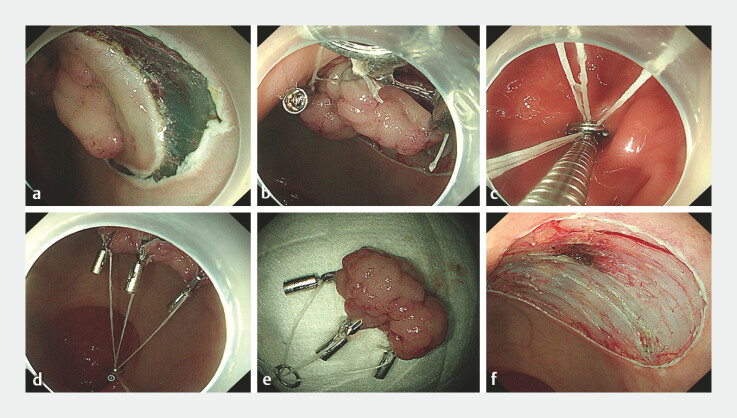
**a** A circumferential incision was performed by submucosal injection at the base of the lesion. **b** At the three equal points along the edge of the lesion, a metal clip carrying dental floss was clamped respectively, and then, three pieces of dental floss were placed in a metal ring outside the body. **c** The metal ring was sent into the body with a metal clip and clamped onto the mucosa on the opposite side of the lesion. **d** Formation of an umbrella-shaped pulley traction structure. **e** Excised gross specimens. **f** The wound surface was clean and smooth.

**Fig. 3:**
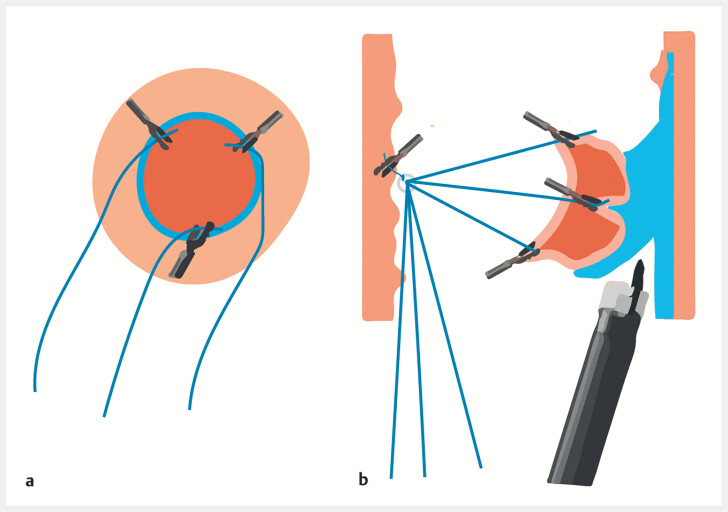
**a** A circumferential incision was performed by submucosal injection at the base of the lesion. **b** Umbrella-shaped pulley traction.

Firstly, a circumferential incision was performed by submucosal injection at the base of the lesion. At the three equal points along the edge of the lesion, a metal clip carrying dental floss was respectively clamped. Subsequently, three pieces of dental floss are placed in a metal ring outside the body. The metal ring was sent into the body with a metal clamp and clamped onto the mucosa on the opposite side of the lesion to form an “umbrella-shaped pulley” structure. The corresponding dental floss can be pulled outside the body as needed to achieve precise traction, allowing the submucosa to be better exposed, providing a clear operational field of view for ESD, and ultimately the lesion is completely removed, ensuring a smooth surgical process ( Fig. 2 , Fig. 3 ).

This “umbrella-shaped pulley” traction overcomes the limitations of the previous unidirectional traction, allowing for multi-point and multi-directional traction, which is more convenient to operate. It helps to quickly, completely and safely separate the lesion, especially suitable for larger areas or lesions with low gravity. It is innovative and worth promoting.

Endoscopy_UCTN_Code_TTT_1AQ_2AD_3AD
